# AAV-mediated gene therapy for metabolic diseases: dosage and reapplication studies in the molybdenum cofactor deficiency model

**DOI:** 10.1186/1479-0556-7-9

**Published:** 2009-06-18

**Authors:** Rita Hahnewald, Waja Wegner, Jochen Reiss

**Affiliations:** 1 Institut für Humangenetik der Universität Göttingen, Heinrich-Düker-Weg 12, 37073 Göttingen, Germany

## Abstract

In a mouse model for molybdenum cofactor deficiency as an example for an inherited metabolic disease we have determined the dosage of recombinant AAV necessary to rescue the lethal deficiency phenotype. We demonstrated long-term expression of different expression cassettes delivered in a chimeric AAV capsid of serotype 1/2 and compared different routes of application. We then studied the effect of double and triple injections at different time points after birth and found a short neonatal window for non-response of the immune system. Exposition with rAAV capsids within this window allows transgene expression after a second rAAV transduction later. However, exposition within this window does not trigger immunotolerance to the viral capsid, which limits rAAV-mediated refurbishment of the transgene to only one more application outside this permissive window.

## Findings

In mammals, molybdenum cofactor (MoCo) is essential for the activity of sulfite oxidase, xanthine dehydrogenase and aldehyde oxidase [1]. The gene products of the human genes *MOCS*1, *MOCS*2, *MOCS*3 and *GEPH *are required for the biosynthesis of MoCo [2]. A mutational block of these genes leads to MoCo deficiency (OMIM #252150) associated with a progressive neuronal damage and death before adolescence in affected patients. The majority of patients suffer from type A deficiency and harbour mutations in the gene *MOCS*1 [3].*Mocs*1 knockout-mice show no detectable residual *Mocs*1 mRNA levels and display a severe phenotype reflecting the biochemical characteristics of human MoCo-deficient patients [4].

Recently, we described the phenotypical rescue of *Mocs*1-deficient mice by intrahepatic injection of a recombinant adeno-associated virus (rAAV) vector carrying an expression cassette for the human *MOCS*1 cDNA [5]. The *MOCS*1 expression cassette has been describe before and essentially contains a hybrid promoter consisting of a cytomegalovirus (CMV) enhancer, a human β-actin promoter, exons 1 through 10 of the human *MOCS*1 gene, a deleted intron 9, which allows for alternative splicing leading to the gene products MOCS1A and MOCS1B and a bovine growth hormone (BGH) polyadenylation (poly A)-signal. MOCS1A and MOCS1B together produce the relatively stable intermediate cPMP, which is further processed to active MoCo by the products of the genes *MOCS*2, *MOCS*3 and *GEPH*.

Transfer of the *MOCS*1 gene was primarily aimed at transduction of hepatocytes, since the liver is the primary organ involved in detoxification of sulfite to sulphate by sulfite oxidase [6]. In the meantime, mice rescued by the intrahepatic rAAV-*MOCS*1 reached a lifespan of up to 666 days. To study the long-term expression profile after AAV transduction, we here analyzed wild-type mice, which had received an intrahepatic injection of AAV encoding the green fluorescent protein (AAV-*EGFP*) on day 6 after birth. The *EGFP *expression cassette contains the coding sequence for *EGFP *instead of the *MOCS*1 cDNA.

**Figure 1:**
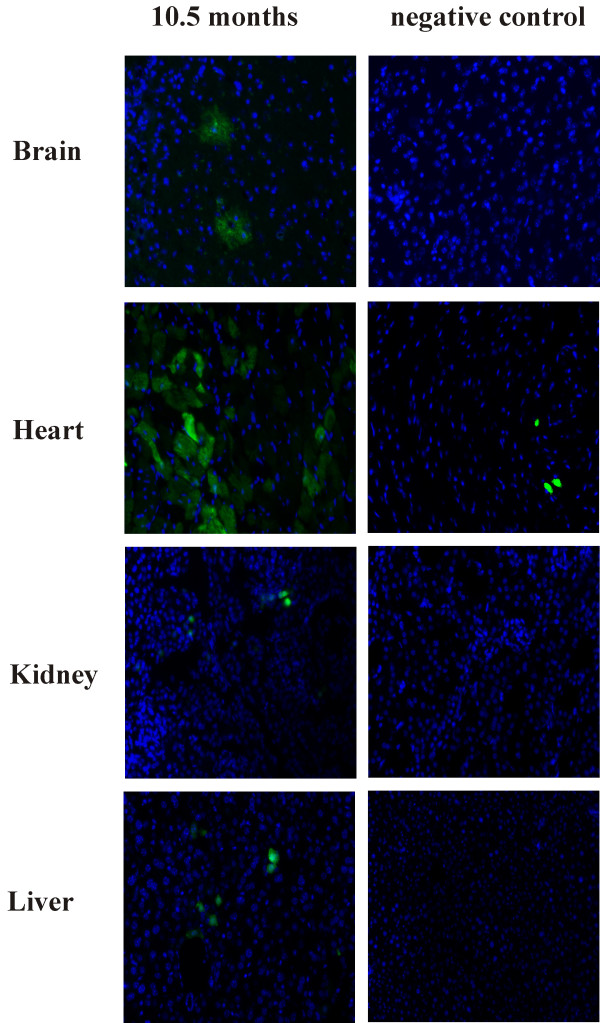
**Long-term expression after AAV1/2 transduction**. Wild-type mice obtained a single intrahepatic AAV-EGFP injection containing 4.5 × 10^9 ^tu on day 6. After 10.5 months the mice were perfused with 4% paraformaldehyde. Tissue for expression analysis was cryoprotected in sucrose and stored frozen at -80° until analysis. Cryostat sections of 3 μm thickness were prepared for EGFP expression analysis. Pictures were recorded by Fluorescent microscope BX60 from Olympus. Fluorescence is shown as an overlay of EGFP (green) and nuclear DAPI (blue) fluorescence. The images were recorded with an exposure time of 50 ms for DAPI and 500 ms for EGFP (1 s for negative expression).

Recombinant viruses were generated by using a mixture of AAV helper plasmids encoding serotype 1 and 2 in ratio of 1:1. Previous studies had demonstrated that the chimeric AAV1/2 vector triggers a higher expression level both in liver and in muscle as compared to serotypes 1 or 2 [7]. Figure 1 shows that the intrahepatic application using AAV1/2 capsids leads to predominant expression in heart and liver, where the transgene product is detectable for more than 10 months. However, the rate of transgene expressing cells drops down from almost 100% in liver [5] to approximately 5%, while in the heart after 10 months still approximately 50% of cells expressed *EGFP *(figure 1). A similar expression profile has been observed in mice carrying the *EGFP *expression cassette in all cells after microinjection of fertilized oozytes and subsequent breeding (data not shown), which indicates that not the rate of transduction but rather persistence of expression accounts for this organ-specific difference.

As a further approach to the treatment of patients, we investigated the efficacy of systemic AAV delivery. Comparative studies 1 month after rAAV-*EGFP *application showed similar tissue transduction after either intrahepatic or intravenous injection [5]. Using the *MOCS*1 expression cassette in an AAV 1/2 capsid, we here studied the effect of systemic delivery by tail vein injections. For this application we used mice with a minimum body weight of around 15 g corresponding to an age of approximately 40 days. Untreated *Mocs*1 deficient mice are unable to build cPMP, the first intermediate in the MoCo biosynthesis, and die on average 7.5 days after birth [4]. We pretreated *Mocs*1-deficient mice until day 40 with periodic intrahepatic injections of purified cPMP from *Escherichia coli *[8] to achieve a suitable size for tail vein injection.

**Figure 2:**
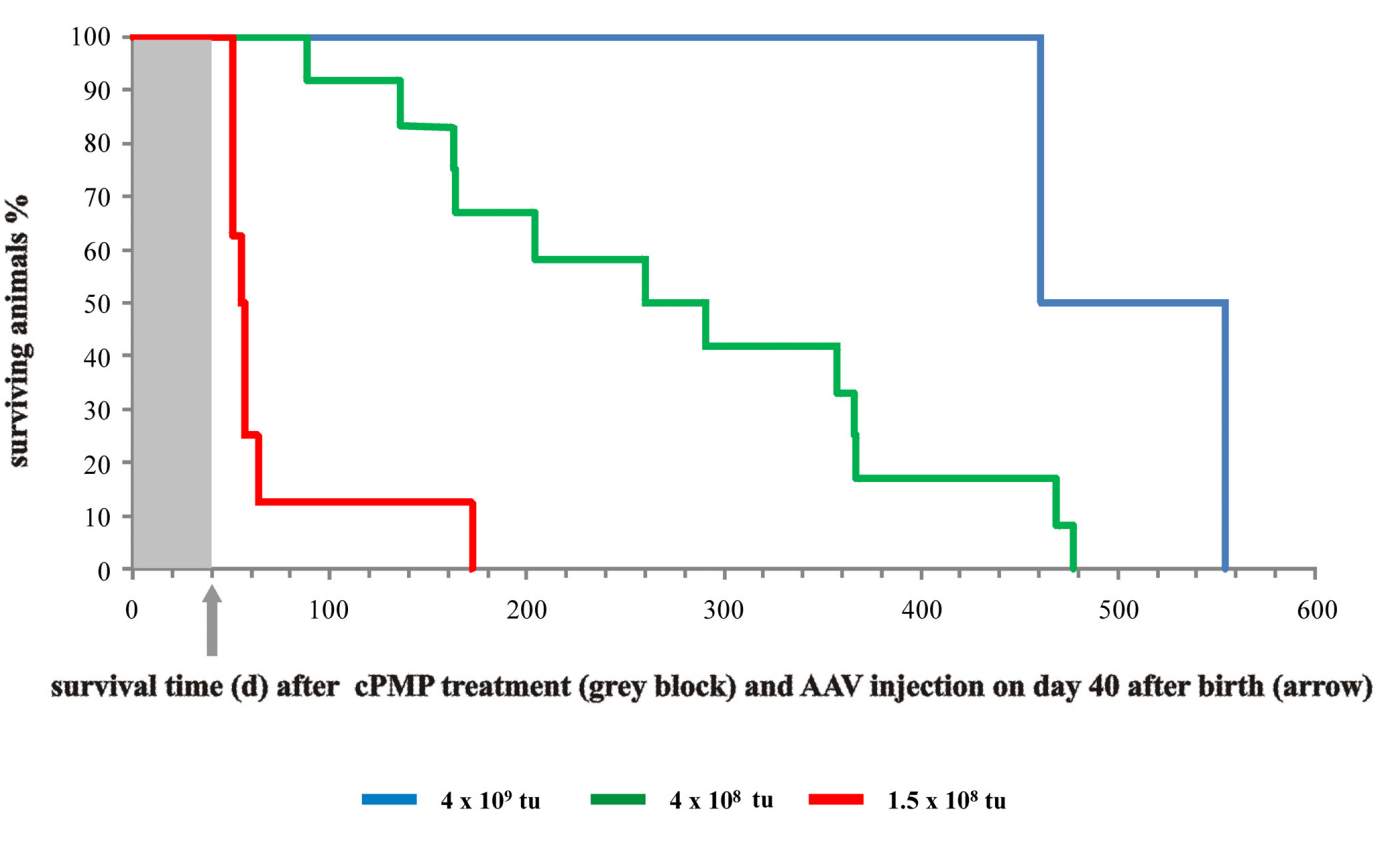
**De-escalations studies of AAV1/2-MOCS1 delivery**. Survival of *Mocs*1-deficient mice injected i.v. on day 40 after birth with various amounts of AAV-*MOCS*1. Group A (blue) was injected with 4 × 10^9 ^tu (n = 2). Group B (red) received 1.5 × 10^8 ^tu AAV-*MOCS*1 (n = 8). Group C (green) received a single injection containing 4 × 10^8 ^tu (n = 12).

Intrahepatic injections were done every other day with increasing amounts from 2 μg in the first week up to 32 μg from the 5th week onward. On day 40, they received 4 × 10^9 ^tu AAV-*MOCS*1 by a single intravenous injection (n = 5). Control animals (n = 10) received the rAAV vector, in which the *MOCS*1 cDNA was replaced by the fluorescent reporter *EGFP *cDNA. These controls died on average 11 days after the final cPMP injection. In contrast, none of the mice treated with AAV-*MOCS*1 died from MoCo deficiency. Two of them have reached a life span of approximately 500 days without cPMP supplementation (figure 2, blue line). The other three mice were sacrificed in the course of reapplication studies (see below).

Considering the lower dosage of 4 × 10^9 ^tu AAV-*MOCS*1 for systemic delivery, as compared to 1 × 10^10 ^tu for the intrahepatic injections described previously [5] and above, the results described here indicate a similar efficacy for both application schemes. All five above described mice had been mated and were fertile. The offspring (n = 64) died on average on day 5.35 after birth, which corresponds to the lifespan of untreated homozygous *Mocs*1 knockout mice from matings of heterozygous mice. This is indirect evidence that the intravenous tail vein injections did not result in germ line transmission of the vector genome.

To estimate the necessary dosage for the treatment of humans, we determined the minimal dosage required to rescue the deficiency phenotype via the intravenous route. 20 neonatal *Mocs*1-deficient mice were pretreated with purified cPMP as described above. At day 40 after birth, the animals obtained a single intravenous tail vein injection containing various amounts of AAV-*MOCS*1 in phosphate-buffered saline. First, we investigated the effect of a thirty-fold reduced dosage of AAV-*MOCS*1 as compared to the experiments described above, i.e. 1.5 × 10^8 ^tu (n = 8). Mice of this group died on average 28.75 ± 6.5 days after the AAV-*MOCS*1 injection and discontinuation of cPMP substitution (figure 2, red line). This reduced dosage apparently is not sufficient to rescue the lethal phenotype.

Next, we studied the effect of an intermediate dosage of 4 × 10^8 ^tu AAV-*MOCS*1 on day 40 after cPMP pre-treatment (n = 12). The mice of this group died on average 238.5 ± 124.4 days after AAV-*MOCS*1 injection and cPMP withdrawal at day 40 after birth (figure 2, green line). All animals of this group were mated and all but one were fertile. Again, the offspring died within the range of untreated animals (data not shown). The observed high variance of the life span suggests that the intermediate dosage of 4 × 10^8 ^tu AAV-*MOCS*1 represents a borderline result and indicates a range for the minimal dosage required for abolishing the MoCo deficiency phenotype. With a maximum body weight of 40 g for the mice used here this would correspond to 1 × 10^10 ^tu per kg body weight. A one year old child with a body weight of 10 kg thus would require 1 × 10^11 ^tu of AAV-*MOCS*1, which is within the range of GMP production facilities.

Although one single injection could abolished the phenotype of the MoCo-deficiency, our murine model allows a prediction only for the natural life span of mice, i.e. 2 to 3 years. In contrast to long-lasting expression in mice, rats, hemophilic dogs and nonhuman primates, expression at therapeutic levels in humans was limited to a period of around 8 weeks [9-14]. This difference was mainly attributed to prior infection of the human patients with natural AAVs in combination with helper adenovirus [15]. This leads to formation of memory CD8^+ ^T cells and their activation upon reexposure to the AAV capsid.

Thus, the possibility of repeated vector administrations in the treatment of patients from an immunological point of view is an important issue to be addressed. To this end, we investigated the feasibility of successful rAAV re-administration at different time points in the MoCo deficient mouse model and compared the reapplication possibilities in different developmental stages. AAV serotype 1/2 (in a 50:50 ratio) was used throughout.

**Figure 3:**
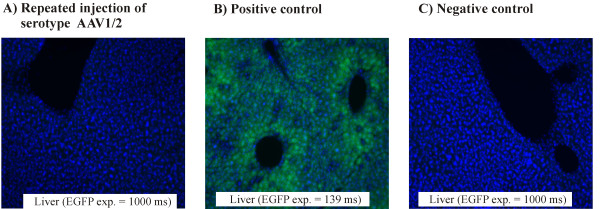
**Vector reapplication in adolescence**. Liver sections of adult mice after different treatment schemes. Exposure times (exp.) for EGFP are indicated (for further details see figure 1). A) First injection i.v. on day 40 with AAV-*MOCS*1, second injection intrahepatic with AAV-*EGFP *after 3 months. B) Only one intrahepatic AAV-*EGFP *injection 4 months after birth. C) No injection.

In the first experiment, two *Mocs*1-deficient mice were pretreated for 40 days after birth with purified cPMP as described above. At day 40, the two animals obtained a single intravenous tail vein injection of 65 μl containing 4 × 10^9 ^tu AAV-*MOCS*1. The successful transduction of this first injection was confirmed by the prolonged lifespan of the otherwise MoCo-deficient animals. The mice were injected for the second time after three months with an AAV vector carrying a reporter gene vector (4 × 10^9 ^tu AAV1/2-*EGFP*). As a positive control for the second injection, two wild-type mice obtained only 4 × 10^9 ^tu AAV1/2-*EGFP*. The negative controls were two wild-mice without any treatment. Two months after AAV1/2-*EGFP *injections, all six mice were perfused with paraformaldehyde. The second AAV injection did not result in any observable expression of *EGFP *in the liver (figure 3).

Studies on hemophilia B mice showed that *in utero *or neonatally AAV-treated mice do not develop antibodies to the AAV capsid after the first injection [16]. They demonstrated that it is possible to establish tolerance to the transgene product human factor IX by these early injections and to obtain long-term therapeutic levels in immunocompetent mice. Here, the transgene products of the *MOCS*1 expression cassette are localized intracellular and thus not accessible for antibodies. We therefore concentrated on the existence of a "window of opportunity" to induce tolerance against the viral capsid in repeated exposures.

**Figure 4:**
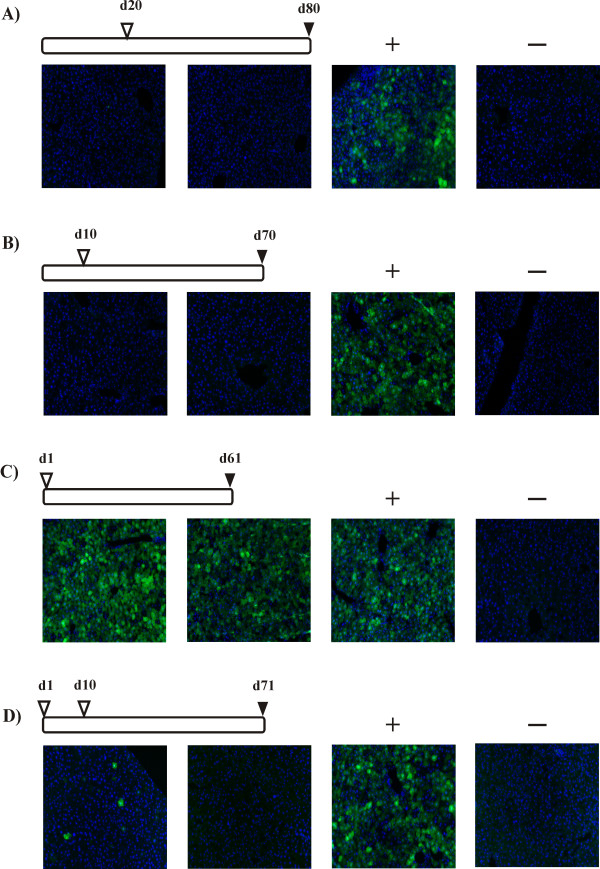
**Reapplication at different developmental stages**. *EGFP *expression two months after AAV-*EGFP *injection (filled triangles) following AAV expositions (open triangles) at different developmental stages. Animals received intrahepatic AAV-*MOCS*1 injections as indicated on top of the lines and an intrahepatic AAV-*EGFP *injection 2 months after the last AAV-*MOCS*1 injection. Liver sections of 2 animals are shown for each time point. Positive control animals (+) received only an AAV-*EGFP *injection. Negative controls (-) received no AAV. Further details are describe in figure 1.

Three groups of two *Mocs*1-deficient mice each received an intrahepatic injection of 50 μl containing 1 × 10^9 ^tu AAV-*MOCS*1 on day 1, day 10 or day 20, respectively. The mice were injected for the second time three months after the first injection with 50 μl containing 1 × 10^9 ^tu AAV-*EGFP*. Two wild-type mice served as negative controls and obtained no second injection. Additionally, for each time point two wild-type mice served as positive control for the AAV-*EGFP *injections and obtained only the second injection with 1 × 10^9 ^tu AAV-*EGFP*. Two months after the AAV-*EGFP *injections, all mice were perfused with 4% paraformaldehyde. The groups with the first injection at day 10 or day 20 the second injection of AAV-*EGFP *did not result in any observable expression of EGFP in the liver (figure 4a, b). In the group injected first at day 1 after birth, both mice showed strong EGFP-expression (figure 4c), which confirms that the immune system shortly after birth does not react to the vector capsid.

Since the products of the *MOCS*1 and the *EGFP *expression cassette do not share cross-reacting epitopes, we could investigate the potential of early injections to induce an immune tolerance against the viral capsid by triple injections. Two wild-type mice obtained a first intrahepatic injection of 1 × 10^9 ^tu AAV-*MOCS*1 on day 1 after birth and a second injection with 1 × 10^9 ^tu AAV-*MOCS*1 on day 10. After two months they received a third injection of 1 × 10^9 ^tu AAV-*EGFP*. A positive control for the AAV-*EGFP *injections obtained only a single injection of 1 × 10^9 ^tu AAV-*EGFP*. Two months after AAV-*EGFP *injections, all mice were perfused with 4% paraformaldehyde. Here, the rAAV-*EGFP *injections did not lead to an *EGFP *expression (figure 4d), even though the first exposure to AAV1/2 capsid occurred on day 1 after birth (compare figure 4c). While the role of a cytotoxic T-cell response in mice remains unclear, the immune system clearly built neutralizing antibodies (nABs) [17,18] against the viral vector after the second injection of viral vector. Thus, the early exposure of the immune system to viral vector capsid allows a successful second application but does not induce an immunotolerance against the capsid proteins.

An important factor in nAB response is the time point of viral vector administration. The group of Petry et al [19]. showed that the efficacy of readministration is dependent on the titer of nAB and that the level of nABs is proportional to the virus dose used for the first injection. Since repeated AAV treatment in adolescence leads to immune responses, future experiments will have to show whether the combination of early first exposure, a lower dosage of virus and/or temporary immunosuppression (e.g. with cyclosporine) facilitates more successful rAAV reapplications.

## Competing interests

The authors declare that they have no competing interests.

## Authors' contributions

RH participated in the design of the study, carried out the practical work and drafted the manuscript. WW participated in the practical work and discussions. JR designed this study and edited the manuscript. All authors read and approved the final manuscript.
